# Fatal Outcome in a Hepatitis E Virus/Human Immunodeficiency Virus Co-Infected Malnourished Child in the Central African Republic

**DOI:** 10.3390/idr12030017

**Published:** 2020-11-12

**Authors:** Stéphanie Judith N’Yetobouko Tabounie, Simplice Cyriaque Kango, Julie Bouscaillou, Vianney Tricou, Arnaud Fontanet, Mirdad Kazanji, Narcisse Patrice Komas

**Affiliations:** 1 Viral Hepatitis Laboratory, Institut Pasteur de Bangui (IPB), Bangui BP 923, Central African Republic; nyetoboukostephanie@yahoo.com (S.J.N.T.); julie.bouscaillou@gmail.com (J.B.); 2 Virology Department, Institut Pasteur de Bangui (IPB), Bangui BP 923, Central African Republic; vianney.tricou@gmail.com (V.T.); mirdad.kazanji@pasteur.fr (M.K.); 3 Centre Hospitalier Universitaire Pédiatrique de Bangui (CHUPB), BP 923 Bangui, Central African Republic; kango.cyriaque@yahoo.fr; 4 Médecins du Monde, 75018 Paris, France; 5 Emerging Diseases Epidemiology Unit, Institut Pasteur, 75015 Paris, France; arnaud.fontanet@pasteur.fr; 6 Conservatoire National des Arts et Métiers (CNAM), 75003 Paris, France; 7 Institut Pasteur de Guyane (IPG), 97300 Cayenne, France

**Keywords:** Central African Republic, HIV/HEV co-infection, hepatitis E virus (HEV), human immunodeficiency virus (HIV), pediatrics, severe acute malnutrition

## Abstract

Hepatitis E virus (HEV) infection is responsible for major endemic outbreaks in developing countries. Human immunodeficiency virus (HIV) and HEV are widespread in the Central African Republic. We report the first documented case of an HEV infection in a 36-month-old child already suffering from HIV and severe acute malnutrition (SAM). The HIV patient was hospitalized for SAM with persistent diarrhea and prolonged fever. The presence of IgG anti-HEV antibodies was noted. Sequencing of the amplified HEV RNA revealed the presence of genotype 3c. The alanine aminotransferase level was slightly above average. The patient died despite being treated by antiretroviral therapy accompanied by probabilistic antibiotic therapy and nutritional rehabilitation. HEV/HIV co-infection in a malnourished patient can accelerate a fatal outcome. In the presence of biological abnormalities in a severe acutely malnourished HIV-infected patient, HEV RNA detection should be added to the standard medical assessment in sub-Saharan African countries.

## 1. Case

**Figure 1:**
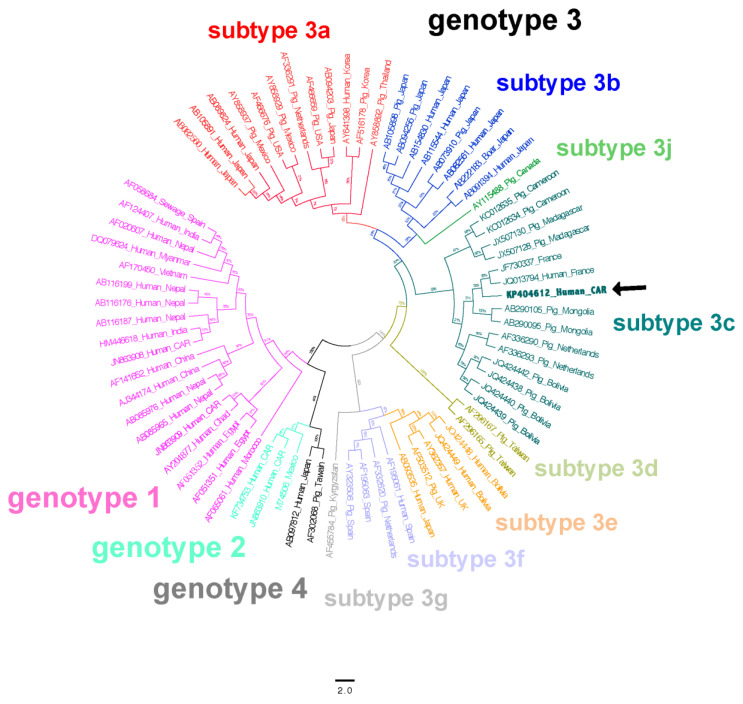
Phylogenetic tree based on partial nucleotide sequence of the hepatitis E virus (HEV) genome. HEV RNA was extracted from the patient’s blood and amplified using RT-PCR. Sequencing was carried out by GATC (Germany).

A 36-month-old female child weighing 8.9 kg and with a height of 82.4 cm was admitted to the Unité Nutritionnelle et Thérapeutique (UNT) of the Centre Hospitalier Universitaire Pédiatrique de Bangui (CHUPB, the main referring pediatric hospital in the Central African Republic (CAR)) for hospitalization due to unexplained severe acute malnutrition (SAM). Clinical and physical examinations upon admission on 16 April 2012 revealed purulent otorrhea in the right ear, which had started approximately nine days earlier, and asthenia with muscle atrophy accompanied by oedema of the lower limbs, in addition to abdominal soreness associated with a diarrheal syndrome for at least the past three weeks. The treatment for SAM was initiated with F75 therapeutic milk during the acute phase followed by F100 therapeutic milk, rehydration with an oral rehydration solution (ReSoMaL), and probabilistic antibiotic therapy with ceftriaxone and amoxicillin to stabilize the health status of the patient. At the same time, HIV and malaria tests were carried out according to the CAR national protocol for malnourished children. The patient was determined to be HIV-seropositive, but the malaria test was negative. The measured T CD4+ cell count was 469 cells/mm^3^. The viral load was not determined. An antiretroviral therapy based on zidovudine/lamivudine/nevirapine (AZT/3TC/NVP) was initiated according to the national CAR protocol for HIV care. The presence of mild conjunctival jaundice and hepatomegaly combined with a slight increase in transaminases led to screening for infections with hepatitis B and C viruses 18 days after admission. These tests were negative. Unpublished results of screening for infection by the hepatitis A virus in the population of children under the age of five attending the Institut Pasteur de Bangui for their biological examinations have shown that 100% of these children had anti-hepatitis A antibodies (IgG), indicative of past contact with the hepatitis A virus. We hence decided to not screen for the presence of hepatitis A virus in this patient. In contrast, ELISA tests (DialPro kit for ELISA test, Italy) revealed the presence of the anti-hepatitis E virus (HEV) antibody (IgG), a marker of a prior infection, but the anti-HEV antibody (IgM), a marker of an ongoing infection, was negative. In addition, the amplification, using real-time and conventional RT-PCR techniques, of HEV RNA from the patient’s blood was positive and sequencing indicated the presence of HEV subtype 3c (GenBank accession number KP404612, Figure 1). Liver enzyme levels were measured using a Pentra 400 device (Horiba, Montpellier, France) and only the alanine aminotransferase (ALT) level was slightly higher than normal. Other parameters, such as the brachial perimeter (116) and the weight/height index (z score of −2), indicated an overall alteration of the health status of the patient. The patient died on 9 May 2012, three weeks after her admission to UNT, following continuous deterioration of her general health status, despite the treatments she received. The general health status of the patient, as well as the clinical and physical parameters, were indicative of stage four of the WHO clinical staging classification for HIV/AIDS. Since malnutrition is often a factor in immune depression, the degree of immunosuppression in this patient is likely to have been particularly severe. The family setting was surveyed and no concomitant infections with the HEV3 genotype were found in any of the other family members following RT-PCR testing; however, several family members were positive in the HEV IgG test. There were free-roaming cats and dogs, domestic pigs, goats, and chickens in and around the house of the patient, but blood and/or stools from these animals could not be taken due to the armed conflict that led to the displacement of populations and the dispersal or slaughter of these animals.

## 2. Discussion

We report here the first description of an HIV/HEV co-infection in a child with SAM. HEV is a major cause of enterically-transmitted acute hepatitis worldwide [1]. Four genotypes have been reported in humans. HEV-1 and HEV-2 are restricted to humans and can cause large water-borne outbreaks in developing countries [2]. These outbreaks usually occur during the rainy season with the oral-fecal route being the most common route of contamination [1,3]. In contrast, HEV-3 and HEV-4 usually result in sporadic human cases in the developed world that are related to zoonotic transmission [4]. HEV-3 is also the main cause of chronic hepatitis in immunosuppressed patients [2]. Although the majority of HEV infections are asymptomatic, symptomatic HEV infections can be severe or even fatal [5,6,7,8,9,10]. HIV is responsible for 7% of deaths in children less than five years of age in Africa [5]. Malnutrition is endemic in many parts of sub-Saharan Africa and contributes to more than 1/3 of deaths in children less than five years of age globally [5]. Malnutrition and HIV infection share similarities in terms of their clinical presentations, with a cycle of infection, malnutrition, and immunodeficiency [6]. Although several studies on the association between HIV and malnutrition have been reported, HIV/HEV co-infection in malnourished children has not been described to date. Malnutrition is one of the main consequences of repeated opportunistic infections and it is a key marker for the accelerated physical deterioration contributing to the excess mortality in HIV-infected children [11,12,13]. 

This case raised the question of the origin of this HEV-3 infection. Although HEV virus is endemic in the CAR, HEV-3 has not been previously reported in this country [8,9,10]. In light of the routes of contamination by HEV-3, it is likely that this 36-month-old girl had been infected by oral contact with food or products contaminated by the waste of domestic animals living in her neighborhood that were infected with HEV-3. Unfortunately, no blood samples or feces samples from the domestic animals living in the immediate vicinity of the patient were collected to confirm the origin of the infection. However, HEV screening in adults living in the same house and in the immediate environment of the patient only revealed the presence of anti-HEV IgG antibodies, while the molecular investigations were negative. 

Another question is the cause of death. It is possible that the HEV infection played a role in the fatal outcome. The patient was put on highly active antiretroviral therapy, with a T CD4+ lymphocyte count above the cut-off of 350 cells/mm^3^ recommended by the WHO, and on nutritional rehabilitation with probabilistic antibiotic therapy, according to national standards [11,12,13]. While the association of HIV infection and malnutrition can be fatal in children, even when treated by multiple antiretroviral therapies, recent studies have shown that HIV-infected children with SAM may respond well to nutritional rehabilitation despite low T CD4+ cell counts [14]. However, the HEV infection could have impaired liver function and exacerbated the hepatotoxic effects of the nevirapine. The patient did not exhibit any clinical manifestations of an HEV infection during her hospitalization, although the ALT level was slightly above normal, which is also often found in people living with HIV. Based on the clinical and physical signs, four hypotheses can be made: (1) an oto-rhino-laryngopharyngeal and digestive infection resistant to antibiotic treatment, with the emergence of resistant germs due to selective pressure, (2) a nosocomial infection, (3) the occurrence of an immune reconstitution inflammatory syndrome (IRIS) in relation to the antiretroviral agents used, or (4) liver failure are related to the HEV infection. Ultimately, without an autopsy or any further investigations, it is not possible to draw conclusions regarding the contribution of the HEV infection to this fatal outcome.

## 3. Conclusions

This study reports the first HIV/HEV co-infection in a child with SAM. Due to the premature death, we could not further investigate the possible complications that could have contributed to the fatal outcome. However, in sub-Saharan African countries with limited resources, in addition to screening for common intestinal parasites, we recommend screening for the routine serological markers of viral hepatitis in HIV-infected children to avoid possible complications due to hepatitis viruses.

## Abbreviations

ALT: alanine aminotransferase; AZT: azidothymidine; CAR: Central African Republic; CNHUPB: Centre National Hospitalier Universitaire Pédiatrique de Bangui; HEV: hepatitis E virus; HEV-1: hepatitis E virus genotype 1; HEV-2: HEV genotype 2; HEV-3: HEV genotype 3; HEV-4: HEV genotype 4; HIV: human immunodeficiency virus; IgG: immunoglobulin G; IgM: immunoglobulin M; IRIS: immune reconstitution inflammatory syndrome; NVP: Nevirapine; ReSoMaL: rehydration solution; RNA: ribonucleic acid; RT-PCR: reverse transcription-polymerase chain reaction; SAM: severe acute malnutrition; 3TC: Lamivudine; UNT: Unité Nutritionnelle et Thérapeutique; WHO: World Health Organization.

## References

[1] Purcell R., Emerson S. (2008). Hepatitis E: An emerging awareness of an old disease. J. Hepatol..

[2] Khuroo M.S., Khuroo M.S., Khuroo N.S. (2016). Hepatitis E: Discovery, global impact, control and cure. World J. Gastroenterol..

[3] Geng Y., Wang Y. (2016). Transmission of Hepatitis E Virus. Adv. Exp. Med. Biol..

[4] Gil Melgaço J., Gardinali N.R., Mello V.D.M.D., Leal M., Lewis-Ximenez L.L., Pinto M.A. (2018). Hepatitis E: Update on Prevention and Control. BioMed Res. Int..

[5] UNICEF: Monitoring the Situation of Children and Women Statistics by Area/Child Nutrition. http://www.childinfo.org/malnutrition.html.

[6] WHO (2008). UNICEF: Integrated Management of Childhood Illness for High HIV Settings.

[7] Rice A.L., Sacco L., Hyder A., Black R.E. (2000). Malnutrition as an underlying cause of childhood deaths associated with infectious diseases in developing countries. Bull. World Health Organ..

[8] I Goumba A., Konamna F.X., Komas N.P. (2011). Clinical and epidemiological aspects of a hepatitis E outbreak in Bangui, Central African Republic. BMC Infect. Dis..

[9] Escribà J.M., Nakouné E., Recio C., Massamba P.-M., Matsika-Claquin M.D., Goumba C., Rose A., Nicand E., García E., Leklegban C. (2008). Hepatitis E, Central African Republic. Emerg. Infect. Dis..

[10] Bouscaillou J., Komas N., Tricou V., Nakouné E., Selekon B., Fontanet A., Kazanji M. (2013). Imported Hepatitis E Virus, Central African Republic, 2011. Emerg. Infect. Dis..

[11] World Health Organization (2006). Antiretroviral Therapy for HIV Infection in Infants and Children in Resource-Limited Settings: Towards Universal Access: Recommendations for a Public Health Approach.

[12] World Health Organization (2008). Antiretroviral Therapy for Infants and Children: Report of the WHO Technical Reference Group, Paediatric HIV/ART Care Guideline Group Meeting.

[13] World Health Organization (2010). Antiretroviral Therapy for HIV Infection in Infants and Children: Toward Universal Access.

[14] Hughes S.M., Amadi B., Mwiya M., Nkamba H., Mulundu G., Tomkins A., Goldblatt D. (2009). CD4 Counts Decline Despite Nutritional Recovery in HIV-Infected Zambian Children with Severe Malnutrition. Pediatrics.

